# Atypical Femoral Fracture in Patients With Metastatic Bone Tumors: An Analysis Based on the Japanese Adverse Drug Event Reaction Database (JADER)

**DOI:** 10.7759/cureus.84126

**Published:** 2025-05-14

**Authors:** Yuka Aimono, Tomiko Sunaga, Ryo Yonezawa, Ayako Tsuboya, Mari Kogo, Shinjiro Tsuge, Akihiro Tamura, Ako Itoh

**Affiliations:** 1 Department of Pharmacy, Hitachi General Hospital, Hitachi, JPN; 2 Clinical Epidemiology, Division of Applied Pharmaceutical Education and Research, Hoshi University, Tokyo, JPN; 3 Department of Hospital Pharmaceutics, School of Pharmacy, Showa Medical University, Tokyo, JPN; 4 Department of Hospital Pharmaceutics, Showa University Northern Yokohama Hospital, Yokohama, JPN; 5 Division of Pharmacotherapeutics, Department of Clinical Pharmacy, Showa Medical University School of Pharmacy, Tokyo, JPN; 6 Department of Orthopedics, Hitachi, Ltd. Hitachi General Hospital, Hitachi, JPN; 7 Department of Breast and Thyroid Surgery, Hitachi, Ltd. Hitachi General Hospital, Hitachi, JPN

**Keywords:** adverse events, atypical femoral fracture, bisphosphonates, bone-modifying agents, denosumab, japanese adverse drug event report (jader) database, long-term efficacy, metastatic bone tumors, reporting odds ratio, zoledronic acid hydrate

## Abstract

Objective

Denosumab (DEN)-related atypical femoral fracture (AFF) is a rare entity, and hence not feasible to examine with a single institution-based study. In light of this, we performed a retrospective analysis of the clinical characteristics of patients with metastatic bone tumors treated with DEN and developed AFF.

Methods

The Japanese Adverse Drug Event Report (JADER) database (2023.8 public version) from the second quarter of 2004 to the second quarter of 2023 was used to investigate the backgrounds of patients with metastatic bone tumors who developed AFF while receiving DEN. The time of AFF onset was defined as the number of days from the start of treatment to the onset of AFF. We also aimed to identify drugs associated with the development of AFF. Cut-off values for signal detection were χ2 ≥4 and number of reports ≥3.

Results

The JADER database contained 2,012 cases of metastatic bone tumors for which DEN was the suspect drug or administered concomitantly with the suspect drug. Of these cases, 106 (5.3%) had AFF, with 91 (85.8%) being women and 61 (57.5%) patients receiving drugs for osteoporosis. The duration from administration to the onset of AFF by DEN was known in 36 cases, and the median value was 926 [interquartile range (IQR): 534-1,552] days. Furthermore, among the drugs suspected of involvement other than DEN, a signal was detected for ZOL, with a reporting odds ratio (OR) of 6.93 and a 95% confidence interval (CI) of 4.39-10.93.

Conclusions

In JADER, AFF in patients with metastatic bone tumors receiving DEN was more common in women and patients receiving osteoporosis drugs, and the time of onset of AFF was approximately 2.5 years.

## Introduction

Bone-modifying agents (BMAs) are used to treat metastatic bone tumors and are also administered to reduce bone loss caused by bone metastases. Although adverse events, such as hypocalcemia and osteonecrosis of the jaw, have been reported, atypical femoral fractures (AFFs) are exceedingly rare. AFF has been associated with the long-term use of bisphosphonates (BPs), but not with denosumab (DEN), an anti-receptor activator of the nuclear factor-κB ligand antibody. The use of BMAs to treat metastatic bone tumors is expected to reduce the frequency of severe skeletal-related events and ameliorate bone pain [1,2]. 

AFF was reported in 2005 as a characteristic femoral fracture in osteoporotic patients receiving long-term treatment with BPs [3]. In 2013, the American Society for Bone and Mineral Research (ASBMR) published diagnostic criteria for AFF [4]. A few studies have recently reported the use of DEN as a single agent to treat metastatic bone tumors [5,6]. The ASBMR Task Force reported that the risk of developing AFF increased with the duration of treatment with BPs [7]. AFF has been associated with the stronger suppression of bone metabolic turnover and a longer bone healing time than general fractures [8], and when cancer patients develop complete fractures, chemotherapy must also be interrupted due to surgery and rehabilitation. Information on the risk of developing AFF during DEN administration may be obtained in advance, which helps in monitoring for AFF and facilitates its prevention and early detection. Since DEN-related AFF is rare, it is not feasible to analyze with a single institution-based study. Hence, we performed a retrospective analysis of the clinical characteristics of patients with metastatic bone tumors treated with DEN who developed AFF.

## Materials and methods

Survey targets 

JADER data provided by the Pharmaceuticals and Medical Devices Agency was used as the database to elicit information on adverse drug reactions. Data extraction was performed by a contractor (Intage Healthcare Inc.). JADER is divided into four tables: patient information data, adverse event report drug and concomitant drug data, adverse event data, and data on the course of adverse events. The data eligible for analysis was defined as all reported data, including adverse event reports, from the second quarter of 2004 to the second quarter of 2023 (2023.8 public version). The study period included all reported data (2023.8 public version), including adverse drug reaction reports from the second quarter of 2004 to the second quarter of 2023, for reported cases in which DEN was administered with the suspected or concomitant drug; those with unknown registration data were excluded.

**Figure 1:**
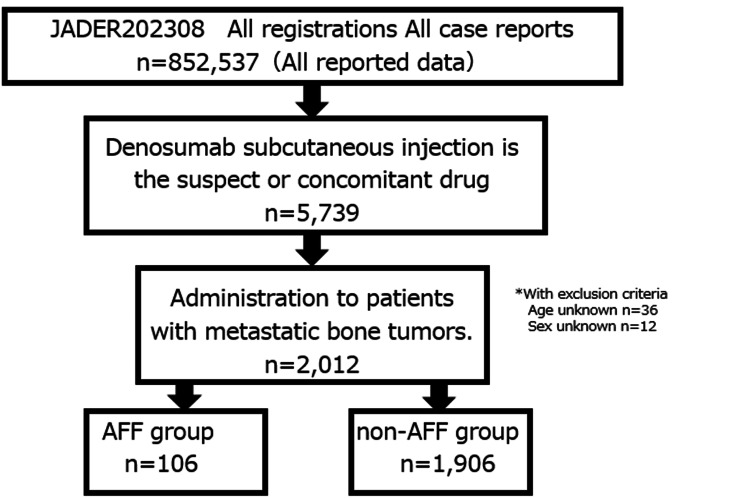
Flowchart of the dataset used in the analysis AFF: atypical femoral fracture; JADER: The Japanese Adverse Drug Event Report

Regarding adverse events in the analysis data, the preferred terms (PT) listed in the ICH International Glossary of Pharmaceutical Terms, Japanese version, and Medical Dictionary for Regulatory Activities, Japanese version (MedDRA/J) ver. 26.0 were used. The AFF group was defined as reports including the term ‘atypical femur fracture (AFF)’ (PT: 10070884), which is the PT listed in MedDRA/J ver. 26.0, while the non-AFF group was defined as all other reports including this term. It is defined as a fracture located along the femoral diaphysis from just distal to the lesser trochanter to just proximal to the supracondylar flare, with at least four of five major features present. These features are as follows: sustained with minimal or no trauma; the fracture line originates at the lateral cortex and is substantially transverse in its orientation (although it may become oblique as it progresses medially across the femur); complete fractures extend through both cortices and may be associated with a medial spike; incomplete fractures involve only the lateral cortex; noncomminuted or minimally-comminuted fractures and localized periosteal or endosteal thickening of the lateral cortex is present at the fracture site. A total of 852,537 reports were registered in JADER, from which data on cases in which DEN was the suspect drug or administered concomitantly with the suspect drug were extracted. A total of 2,012 cases were analyzed, excluding those with no information on age or sex. Of these cases, 106 (5.3%) were in the AFF group and 1,906 (94.7%) were in the non-AFF group (Figure 1).

Survey items

Patient Background

Based on the data analyzed, patient sex and age registered in JADER were investigated as the patient background of the subject reports. Age categories are registered in units of 10 years and terms of adults and older individuals. In the present study, cases were divided into the following two groups: 60+ and elderly, and 20s-50s and adults. In addition, patients with no information on sex or age were excluded.

Time to the Onset of AFF

The number of days until the onset of AFF was investigated, with the first day of treatment being set as day one. Patients with no data on the date of the onset of adverse drug reactions or those with the onset of adverse drug reactions before the start of treatment were excluded.

Extraction of Data on Drugs Associated With AFF

In addition to the 2,012 cases in which DEN was the suspect drug or administered concomitantly with the suspect drug, in patients with metastatic bone tumors, ZOL, steroids, and proton pump inhibitors (PPIs) [9], which have been indicated to increase the risk of AFF, were also included for comparison. In this case, one report of the suspected or concomitant use of DEN was considered. Data were categorized and tabulated for each drug effect.

Statistical Analysis

A 2 × 2 contingency table was created using an imbalance analysis [10], which is used to examine relationships between combinations of medicinal products and adverse events in spontaneous adverse drug reaction report data, and the reporting odds ratio (ROR) and its 95% confidence interval (CI) were calculated. In accordance with reporting [10], cut-off values for signal detection were χ2 ≥4 and number of reports ≥3. SPSS Statistics ver. 27 (IBM Corp., Armonk, NY) was used for statistical analyses.

## Results

Subject reports

Patient Background

In the AFF group, 91 (85.8%) women and 61 (57.5%) patients receiving osteoporosis medications had a higher percentage of reports (Table 1).

**Table 1:** Patient characteristics AFF: atypical femoral fracture

Variable		AFF (n=106), n (%)	Non-AFF (n=1,906), n (%)	Total (n=2,012), n (%)
Sex	Female/male	91/15 (85.8/14.2)	946/960 (49.6/50.4)	1,037/975 (51.5/48.5)
Age	Older than 60 years and elderly/younger than 60 years and adult	66/40 (62.3/37.7)	1,481/425 (77.7/22.3)	1,547/465 (76.9/23.1)
Medication for osteoporosis	With/without	61/45 (57.5/42.5)	383/1,523 (20.1/79.9)	444/1,568 (22.1/77.9)

Time to the Onset of AFF

Of the 106 cases in the AFF-onset group, 36 were included in the study. Breast cancer was the most common primary disease for metastatic bone tumors, accounting for 20 cases (55.6%). The median time to the onset of AFF was 926 [interquartile range (IQR): 534-1,552] days.

Relationship Between AFF and Drugs Other Than DEN

Of the 2,012 cases in which DEN was the suspect drug or administered concomitantly with the suspect drug in patients with metastatic bone tumors, 60 drugs other than DEN were classified as ‘suspect’ for their involvement in AFF. Of these, the ROR and 95% CI of ZOL, steroids, and PPIs are shown in Table 2. The only drug for which a signal was detected was ZOL with ROR of 6.93 (4.39-10.93).

**Table 2:** Identification of drugs associated with AFF ^*^Methylprednisolone, prednisolone, betamethasone, and dexamethasone. ^**^Omeprazole, lansoprazole, rabeprazole, esomeprazole, and vonoprazan AFF: atypical femoral fracture; CI: confidence interval; PPIs: proton pump inhibitors; ROR: reporting odds ratio

Pharmaceutical agent	AFF (n=106), n (%)	Non-AFF (n=1,906), n (%)	Total (n=2,012), n (%)	ROR	95% CI
Zoledronic acid hydrate	32 (30.2)	112 (5.9)	144 (7.2)	6.93	(4.39-10.93)
Steroids^*^	9 (8.5)	350 (18.4)	359 (17.8)	0.41	(0.21-0.83)
PPIs^**^	7 (6.6)	396 (20.8)	403 (20.0)	0.27	(0.17-1.27)

## Discussion

There have been no previous reports using JADER regarding AFF caused by DEN. Therefore, we believe the findings of this study provide useful information in terms of starting DEN treatment. The time of the onset of AFF was 2.5 years, and a signal was detected for ZOL after the extraction of drugs associated with AFF. The availability of relevant information on AFF to patients starting treatment with DEN may lead to its early detection.

As shown in Appendix 1, according to the survey of AFF 2021 registered cases compiled in Japan by the Osteoporosis Committee of the Japanese Orthopaedic Association, 600 (92.2%) cases of AFF (n=651) were female [11]. This is consistent with the results of the JADER survey. In addition, in the final summary of the Specific Use Results Survey on the long-term use of DEN (July 2020), all four (0.11%) AFF cases among those analyzed (n=3,506) were female [12]. Furthermore, the suspect drug was DEN, and adverse events were extracted using the FDA Adverse Event Reporting System with AFF. Data were available for 158,894 reports (up to March 2024). Information was missing on sex, age, reason for use, and osteoporosis, which resulted in their exclusion from further analysis in 6,597 cases. Of these, 112 (1.7%) had AFF and 99 (88.4%) were female [13] (Appendix 1). Femoral diaphyseal fracture is more prevalent in females due to the curvature of the femur, suggesting a relationship between the prevalence of AFF and female sex.

Adults 65 years and older are generally more likely to develop adverse events when administered BMAs compared to younger adults. A previous study reported that bone resorption exceeded bone formation, and bone strength decreased with age in both men and women [14]. On the other hand, since AFF appears to be associated with a younger age than non-AFF [15], the development of AFF needs to be considered irrespective of age. According to the findings of the AFF 2021 registry case study, 413 (63.4%) of AFF cases (n=651) were treated with BPs [11]. Shane et al. have reported that 291 (93.9%) of patients with osteoporosis and concurrent AFF were being treated with BPs [4] (Appendix 1). When BPs are used to treat bone metastases, a careful follow-up is required because, as with osteoporosis, AFF may occur.

According to the special drug results survey, the long-term use of DEN entailed 103-575 days [12]. Therefore, data from the JADER registry showed that AFF occurred after the long-term use of DEN. The mechanisms underlying the onset of AFF are considered to involve the suppression of bone metabolic turnover due to the long-term use of BMAs. In clinical practice, the long-term use of DEN to prolong the survival of cancer patients is also assumed to be a factor contributing to the development of AFF. Regarding the duration of BPs to treat osteoporosis, according to the findings of the AFF 2021 registry case survey, the most commonly observed duration of BPs was longer than three years in 308 (74.6%) cases [11] (Appendix 1). In the study by Shane et al., the average duration of treatment with BPs was seven years [16].

Schilcher et al. showed that the risk of AFF increased with the duration of treatment with BPs, with an accelerated increase from four years onwards [17]. Dell et al. have reported a rate of 1.78/100,000 patients with AFF less than two years after the initiation of DEN treatment and 107.5/100,000 patients after more than 10 years of treatment [18]. On the other hand, AFF was previously shown to occur after 1.9 years [19], two to four years [5,6], 3.5 years [20], and 68-103 (mean: 83.3) months [21] of treatment with BPs for bone metastases (Appendix 2). One-year dose intensities for ZOL and DEN to treat bone metastases were approximately 10- and 12-fold higher, respectively, than those for osteoporosis. Therefore, the time to the onset of AFF after treatment for bone metastases is expected to be shorter.

The findings of a recent follow-up study on DEN in patients with osteoporosis showed that bone density continued to increase when DEN was administered continuously for six years. Furthermore, even after two years of treatment and one year of withdrawal, bone density was the same as that after six years of continuous treatment with DEN. Moreover, bone density remained higher after two years of treatment with DEN and two years of withdrawal than before treatment with DEN [22]. Therefore, DEN may be temporarily withdrawn or the dosing interval may be adjusted. However, we recommend its continuous administration for the first two years. The effects of a gradual reduction in BMAs (mainly 12 weeks of ZOL) were examined in breast cancer patients with bone metastases [23]. In the future, it may be necessary to change (extend) the dosing interval of BMAs [24] (Appendix 2).

ZOL was the main treatment before the launch of DEN. Lockwood et al. indicated the importance of considering the potential complications associated with the use of BPs to accurately diagnose and treat AFF promptly, even in cancer patients [25]. The FDA issued guidance in 2010, stating that patients receiving BPs or DEN (particularly those on long-term treatment for three to five years) need to be instructed to report AFF symptoms and that doctors need to regularly check for these symptoms [26]. Kaku et al. examined 529 patients with metastatic bone tumors who were treated with ZOL or DEN at a single center, and five patients (0.9%) who developed AFF had received ZOL [27].

The incidence of AFF caused by DEN in patients with metastatic bone tumors was 0.4% in a retrospective observational study [28]. However, no randomized trials or other studies have been conducted on DEN-related AFF in patients with metastatic bone tumors. Therefore, further investigations are needed on the frequency of AFF in relation to age and dosages, as well as on the establishment of appropriate dosing and withdrawal periods. We suggest a reassessment of the risk of fracture in female patients with a history of osteoporosis medication or ZOL use from two years after the initiation of DEN and periodically thereafter. Furthermore, if thickening of the lateral femoral cortex is observed, regular bone resorption marker measurements and radiographic examinations may help prevent AFF.

This study has two limitations that need to be addressed. There was a reporting bias, and the amount of individual clinical information available was limited. Since it was not possible to confirm a causal relationship, caution needs to be exercised when referring to the present results for the monitoring of adverse events in clinical practice. If the possibility of an adverse event is indicated, a case-control study is considered necessary to test the hypothesis that a relationship exists between the drug and the adverse event.

## Conclusions

Although AFF is rare, it significantly impacts the quality of life and the activities of daily living. An explanation of AFF to patients and the monitoring of adverse effects are essential for its prevention. In JADER, the incidence of AFF in patients with metastatic bone tumors receiving DEN was higher in women and patients administered osteoporosis drugs. The time of the onset of AFF was approximately 2.5 years, and a signal was detected for ZOL. Further investigations are needed to identify risk factors for AFF. The clinical characteristics identified in the present study may help contribute to the prevention and early detection of AFF.

## Appendices

Appendix 1

**Table 3:** Information on patients with AFF* ^*^[11-14] BP: bisphosphonate; NA: not available

Variables		AFF (n=651), n (%)	AFF (n=4), n (%)	AFF (n=112), n (%)	AFF (n=310), n (%)
Sex	Female/male	600/51 (92.2/7.8)	4/0 (100.0/0.0)	99/13 (88.4/11.6)	NA
Age	Older than 60 years and elderly/younger than 60 years and adult	NA	NA	NA	NA
Medication for osteoporosis	With/without	413/238 (63.4/36.6)	3/1 (75.0/25.0)	NA	291/19 (93.9/6.1)
Duration of BP use	More than 3 years/less than 3 years	308/105 (74.6/25.4)	0/3 (0/100.0)	NA	NA

Appendix 2

**Table 4:** Summary of studies on AFF AFF: atypical femoral fracture; ASBMR=American Society for Bone and Mineral Research; BMD: bone mineral density; BP: bisphosphonate; CI: confidence interval; IVBP: intravenous bisphosphonates; NA: not available; NSCLL: non-small cell lung cancer; RR: relative risk; ST/FS: subtrochanteric/femoral diaphysis

Study	Year	Design	ST/FS, n	AFFs, n (%)	AFFs on BPs, n (%)	Incidence rate	Comments
Shane et al., 2014, USA [16]	2014	Review	NA	NA	NA	The first ASBMR task force reviewed the literature on 310 cases of AFFs, 286 in patients treated with BPs for osteoporosis	Most cases were women and had received oral alendronate monotherapy, although the specific BP was not provided in one‐third of cases. The median duration of BP therapy was 7 years
Schilcher et al., 2015, Sweden [17]	2008-2010	Retrospective cohort case-control	5,475	172 (3)	134 (78)	Among BP users, women had a 3-fold higher risk than men (RR: 3.1, CI: 1.1–8.4). RR after 4 years or more of use reached 126 (CI: 55–288), with a corresponding absolute risk of 11 (CI: 7–14) fractures per 10,000 person-years of use	The risk decreased by 70% per year since the last use
Dell et al., 2012, USA [18]	2007–2011	Prospective cohort incidence	4,036	142 (4)	128 (90)	The age‐adjusted incidence of AFFs with the use of BPs was 1.78/100,000 (1.5–2.0) with 0.1–1.9 years and 113.1/100,000 (69.3–156.8) with 8–8.9 years	For comparison, the incidence of all hip fx in BP‐exposed patients at KPSC was 463/100,000 patients/year in those on BPs for 0–1 years; the incidence of all hip fx decreased on BPs up to 5 years (384, 367–400), then stabilized, and slightly increased after 8–9 years (544/100,000; 522–565
Chang et al., 2012, USA [19]	2005-2010	Retrospective cohort	NA	Breast cancer: 4; multiple myeloma: 2	NA	Duration of treatment with BPs (mo): 68, 93, 103, 69	The median number of IVBP doses was 20 [interquartile range (IQR): 6–41], spanning a median period of 1.9 years
Austin et al., 2016, USA [5]	2016	Case report	NA	NSCLL and breast cancer: 1; prostate cancer: 1	NA	Duration of treatment with BPs (yrs): 2, 3.5	These patients developed AFF 2, 3, and 3.5 years after the initiation of denosumab therapy. These time intervals before fracture are longer than in previous studies, which noted fractures after 1 week to 18 months of treatment
Sugihara et al., 2018, Japan [6]	2018	Case report	NA	Breast cancer: 1	NA	Duration of treatment with BPs (yrs): 4	The patient had no history of BP use
Takahashi et al., 2019, Japan [21]	2012-2017	Retrospective cohort	277	5 (2)	5 (80)	Corresponding to an incidence of 1.8%	Fisher’s exact test was used to evaluate the relationship between potential risk factors and the risk of AFF, and showed that the number of doses of denosumab and prior zoledronic acid treatment correlated with the risk of developing AFF (p=0.0057 and 0.0151, respectively)
Puhaindran et al., 2011, USA [21]	2004-2007	Retrospective cohort	327	4 (1)	4 (100)	Duration of treatment with BPs (mo): 45, 47, 45, 46	There was no significant difference between patients who developed AFF and those who did not, with regard to the doses of IVBP or the duration of treatment
Lacey et al., 2012, USA [22]	2012	Review	NA	NA	NA	NA	An extension study of the multi-armed dose-ranging phase II denosumab study. The model-adjusted mean (95% CI) percent change from the baseline in lumbar spine BMD over 6 years demonstrated that long-term denosumab treatment (60 mg Q6m) continued to augment BMD, with no plateau being observed
Amadori et al., 2013, Italy [23]	2006-2010	Prospective cohort	NA	NA	NA	NA	There was no significant difference between patients who developed AFF and those who did not, with regard to the doses of IVBP or the duration of treatment
Himelstein et al., 2017, USA [24]	2009-2012	Randomized open-label clinical trial	NA	NA	NA	NA	Among patients with bone metastases due to breast cancer, prostate cancer, or multiple myeloma, the use of zoledronic acid every 12 weeks compared with the standard dosing interval of every 4 weeks did not result in an increased risk of skeletal events over 2 years. This longer interval may be an acceptable treatment option
